# No evidence of a synergistic effect of HIV infection and diabetes mellitus type 2 on fat distribution, plasma adiponectin or inflammatory markers

**DOI:** 10.1186/s12879-020-05594-3

**Published:** 2020-11-25

**Authors:** Malene Hove-Skovsgaard, Julie Abildgaard, Marco Gelpi, Julie Christine Gaardbo, Lilian Kolte, Henrik Ullum, Marius Trøseid, Birgitte Lindegaard, Susanne Dam Nielsen

**Affiliations:** 1 Department of Infectious Diseases, Rigshospitalet, Copenhagen University Hospital, Copenhagen, Denmark; 2 The Centre of Inflammation and Metabolism and the Centre for Physical Activity Research, Rigshospitalet, University of Copenhagen, Copenhagen, Denmark; 3 Department of Infectious Diseases, Hvidovre Hospital, University of Copenhagen, Hvidovre, Denmark; 4 Department of Pulmonary- and Infectious Diseases, Nordsjællands Hospital, University of Copenhagen, Hillerød, Denmark; 5 Department of Clinical Immunology, Rigshospitalet, Copenhagen University Hospital, Copenhagen, Denmark; 6 Section of Clinical Immunology and Infectious Diseases, Oslo University Hospital, Oslo, Norway; 7 Research Institute of Internal Medicine, Oslo University Hospital, Oslo, Norway; 8 Institute for Clinical Medicine, University of Oslo, Oslo, Norway

**Keywords:** HIV infection, Diabetes mellitus type 2, Fat distribution, Adiponectin, IL-6, sCD14

## Abstract

**Background:**

Altered fat distribution and chronic inflammation are found in both persons living with HIV (PLWH) and persons with diabetes mellitus type 2 (DM2) and are known risk factors for cardiovascular diseases (CVD). We aimed to investigate if a synergistic effect of HIV infection and DM2 was found on fat distribution and inflammation.

**Methods:**

A cross-sectional study was performed including PLWH with HIV RNA < 200 copies/mL (18 with DM2 (HIV + DM2+), 18 without DM2 (HIV + DM2-)) and controls (19 with DM2 (controls with DM2) and 25 without DM2 (healthy controls). We measured fat distribution using dual-energy X-ray absorptiometry scan. Plasma concentrations of adiponectin, interleukin-6 (IL-6), tumor necrosis factor-alfa (TNF- *α*) and soluble CD14 (sCD14) was measured using snap-frozen plasma.

**Results:**

HIV + DM2+ and HIV + DM2- had comparable trunk/limb fat ratio. In contrast, HIV + DM2+ had a higher trunk/ limb fat ratio than controls with DM2 and healthy controls (*p* = 0.013 and *p* < 0.001, respectively). However, HIV + DM2+ and controls with DM2 had comparable amount of trunk fat mass (kg) (*p* = 0.254). A lower concentration of plasma adiponectin and higher concentration of IL-6 was found in HIV + DM2+ than in HIV + DM2-(*p* = 0.037 and *p* = 0.039) and in healthy controls (*p* = 0.001 and *p* = 0.012). In contrast, plasma adiponectin and IL-6 concentrations were comparable in HIV + DM2+ and controls with DM2 (*p* = 0.345 and *p* = 0.825). Concentration of sCD14 was comparable in HIV + DM2+ and HIV + DM2–(*p* = 0.850), but elevated in HIV + DM2+ compared to controls with DM2 (*p* < 0.001) and healthy controls (*p* = 0.007). No statistical interactions were found between HIV infection and DM2 for any of the depending variables.

**Conclusion:**

A synergistic effect of HIV and DM2 was not found for any of the outcomes. However, HIV + DM2+ had features related to both HIV infection and DM2 with a high trunk/limb ratio, high trunk fat mass, low concentration of plasma adiponectin and elevated concentrations of IL-6 and sCD14. This could contribute to elevated risk of CVD.

## Background

After the introduction of combination antiretroviral treatment (cART), morbidity and mortality related to acquired immune deficiency syndrome (AIDS) have decreased [1]. However, a gap in life expectancy remains [2], and new challenges related to non-AIDS comorbidity have emerged including excess risk of cardiovascular disease (CVD) [3, 4].

Altered body composition with redistribution of body fat from subcutaneous departments to the visceral department is recognized as a common complication in persons living with HIV (PLWH) and especially in those exposed to older generations of cART including thymidine analogues [5]. Recently, weight gain and obesity in PLWH on modern cART regimes have been recognized as an increasing problem [6]. As in the general population weight gain and obesity is associated with both CVD and DM2 in PLWH [7, 8].

Adiponectin is an adipokine with anti-inflammatory properties. Both lipoatrophy and central fat accumulation have been associated with lower concentrations of plasma adiponectin in PLWH [9], and adiponectin could be a link between altered fat accumulation and DM2. Thus, low plasma adiponectin concentration has been associated with increased risk of DM2 in the general population [10], and CVD in both PLWH and in uninfected persons with DM2 [11, 12].

Human immunodeficiency virus infection is characterized by chronic inflammation, and even in persons with undetectable viral replication evidence of inflammation is found, when compared to uninfected controls [13, 14]. Likewise, DM2 is associated with increased inflammation [15].

The aim of this study was to investigate the combined effect of HIV and DM2 on fat distribution and inflammation. We hypothesized that PLWH with DM2 (HIV + DM2+) would have altered fat distribution with more trunk fat mass and less limb fat mass than PLWH without DM2 (HIV + DM2-), controls with DM2, and healthy controls. Also, we hypothesized that plasma adiponectin concentration would be lower and concentrations of IL-6, TNF-*α*, and sCD14 would be higher in HIV + DM2+ than in the three control groups indicating a synergistic effect of HIV infection and DM2.

## Methods

### Participants

This cross-sectional study was a part of a larger study entitled “HIV-infected persons with type 2 diabetes show evidence of endothelial dysfunction and increased inflammation” concerning inflammation and endothelial function in PLWH with DM2 [16]. Participants were included between August 2012 and August 2013. In total 100 participants were included in the primary study. When included all participants were invited to a Dual-energy X-ray absorptiometry scan (DXA-scan) which were optional since the participants had to make an additional visit to the hospital for this scan. In total 80 participants agreed to this (18 with HIV + DM2+, 18 with HIV + DM2-, 19 controls with DM2 and 25 healthy controls). All 80 participants with a DXA-scan were included in the present study. This was a part of a larger study and the present study was designed as an exploratory study. Thus, a power calculation was not done for outcomes presented in this study.

Inclusion criteria for the present study was participation in the study “HIV-infected persons with type 2 diabetes show evidence of endothelial dysfunction and increased inflammation” [16] and willingness to participate with a DXA scan. For the study “HIV-infected persons with type 2 diabetes show evidence of endothelial dysfunction and increased inflammation” the following inclusion criteria were used: Inclusion criteria for PLWH were treatment with cART and undetectable viral replication (defined as HIV RNA < 200 copies/mL). Inclusion criteria for persons with DM2 were confirmed DM2 by a trained clinician and one or more of the following: HbA1c ≥ 48 mmol/mol, fasting venous plasma glucose > 7 mmol/l, or 2-h venous plasma glucose concentration on ≥11.1 mmol/L after a glucose tolerance test prior to this study [17, 18]. All persons with DM2 were treated with diet and/or oral anti-diabetics and/or insulin. All persons without DM2 were required to have both normal fasting venous plasma glucose (< 6.1 mmol/L) and HbA1c < 48 mmol/mol. We did not have to exclude any participants without DM2 included in the study due to elevated plasma glucose level or HbA1c.

Exclusion criteria were immunosuppressive treatment, acute infections, malignancy, and pregnancy. However, no females in the reproductive age group were included in the study. Both written and oral information was given to the study participants prior to inclusion in the study. This included instructions about at least 10-h fasting before time of blood sampling.

All PLWH with DM2 attending routine control for HIV infection at the Department of Infectious Diseases at University Hospital of Copenhagen, Rigshospitalet or Hvidovre Hospital, and who fulfilled inclusion and exclusion criteria for the study “HIV-infected persons with type 2 diabetes show evidence of endothelial dysfunction and increased inflammation” [16], were invited to participate until *n* = 25 were recruited in this group. Inclusion of patients and controls in the other groups was done to achieve best possible match on age and sex. Persons with DM2 were included from the Department of Endocrinology and Centre of Inflammation and Metabolism, University Hospital of Copenhagen, Rigshospitalet. Healthy controls were recruited among hospital staff. All PLWH included in the study had a confirmatory positive HIV test. A negative HIV test was not performed for participants in the uninfected control groups, since the prevalence of HIV in Denmark is 0.1%, and it seems reasonable to assume that clinically healthy persons are HIV-negative. Six controls with DM2 also participated in a study concerning the effect of short duration, high-intensity interval training on endothelial function and metabolism [19]. These participants were included before training.

The study was performed in accordance with the Declaration of Helsinki and approved by the local ethical committee on health research ethics (H-4-2012-076 CIM VEK) and the Danish Data Protection Agency.

### Dual-energy X-ray absorptiometry scan

A DXA–scan was used to measure total fat mass, limb fat mass, and trunk fat mass in all participants (Lunar Prodigy Advance; GE Medical Systems Lunar, Milwaukee, WI, USA). Prodigy Software (enCORE 2004, version 8.8, GE Lunar Corp., Madison, WI, USA).

### Laboratory analyses

Fasting venous blood samples were collected from all participants. HIV RNA was measured in PLWH, and glucose, HbA1c, triglyceride, cholesterol, HDL, LDL, c-peptide, CD4+, and CD8+ counts were measured in all participants as routine analyses at the time of inclusion at the Department of Clinical Biochemistry at Rigshospitalet, Denmark. Tubes with sodium fluoride and potassium oxalate were used to obtain plasma for glucose analyses and tubes with lithium heparin separator were used to obtain plasma for triglyceride, cholesterol, HDL, LDL an c-peptide analyses. The samples were centrifuged within 4 h. Tubes with K_2_EDTA were used to collect full blood for HbA1c analyses. All tubes were from Greiner Bio-One, Kremsmünster, Austria.

Concentrations of plasma adiponectin, IL-6 and TNF- *α* were measured in snap-frozen plasma. Adiponectin was measured using sandwich immunoassay, Human adiponectin Kit (MSD, Gaithersburg, MD, USA). IL-6 and TNF- *α* were measured using a sandwich immunoassay, Proinflammatory Panel 1 (MSD, Rockville, MD, USA). sCD14 was measured in snap-frozen plasma using an enzyme-linked immunosorbent assay (R&D, Minneapolis, USA). All assays were performed according to manufacturers’ instructions.

### Insulin resistance

To measure insulin resistance, we used the Homeostatic Model Assessment of Insulin Resistance (HOMA-IR) [20]. HOMA-IR was calculated using HOMA Calculator (https://www.dtu.ox.ac.uk/ToolsSoftware/) including fasting glucose and C-peptide.

### Statistics

Data were tested for normal distribution, and all soluble markers were logarithmic transformed to obtain normal distribution. Results are given as mean and 95% Confidence Interval (95% CI) or geometric mean (95% CI). Differences between groups were analyzed using one-way ANOVA followed by t test. Main outcomes and possible interactions between HIV infection and DM2 were further investigated using multivariate linear regression. The soluble markers: adiponectin, IL-6, and TNF-α were correlated to fat mass in kg to assess their correlation to fat distribution. Only significant correlations were reported. Correlations were done using Pearson correlation. Pearson chi-square test was used on categorical data. Two-tailed *p*-values < 0.05 were considered significant Statistical analyses were performed using SPSS version 25 (SPSS, Inc.; Chicago, IL, USA), GraphPad Prism 5 (GraphPad Software, San Diego, CA, USA) and R version 3.5.2.

## Results

### Study population

Characteristics of the study population are shown in Table 1. The groups were similar regarding age and sex. There was no difference in treatment duration between the two PLWH groups. HOMA-IR was comparable in HIV + DM2+ and controls with DM2 but elevated compared to HIV + DM2- and healthy controls.

**Table 1:** Clinical characteristics of the study population

	HIV + DM2+	HIV + DM2-	Controls with DM2	Healthy controls	*P*
*n*	18	18	19	25	
Age, median (range)	58 (46–65)	57 (38–67)	57 (49–66)	58 (42–66)	0.801
Gender (%male)	89	94	68	88	0.125
BMI	27 (25–29)	25 (22–27)	29 (27–30)	25 (24–26)	0.003
*Metabolic factors*					
Fasting –PG	8.6 (7.2–10.0)	5.2 (5.0–5.4)*	8.5 (7.5–9.6)	5.3 (5.1–5.5)*	< 0.001
Hba1c (mmol/mol)	49 (44–55)	35 (33–37)*	55 (49–60)	37 (36–38)*	< 0.001
Cholesterol	4.5 (4.1–4.9)	5.6 (5.0–6.1)*	4.2 (3.8–4.7)	5.5 (5.1–5.9)*	< 0.001
HDL	1.1 (0.9–1.4)	1.4 (1.2–1.6)	1.3 (1.1–1.4)	1.5 (1.3–1.7)*	0.010
LDL	2.3 (1.9–2.7)	3.4 (2.8–4.0)*	2.4 (1.9–2.8)	3.5 (3.2–3.8)*	< 0.001
Triglyceride	2.7 (1.8–3.7)	2.0 (1.2–2.8)	2.0 (1.5–2.5)	1.4 (1.0–2.0)*	0.006
HOMA-IR	3.3 (2.6–3.9)	2.0 (1.6–2.3)*	3.3 (2.5–4.1)	1.7 (1.4–2.0)*	< 0.001
Fasting c-peptide, median (range)	0.96 (0.58–1.44)	1.09 (0.66–2.18)*	1.05(0.39–2.32)	0.75 (0.39–1.42)*	< 0.001
*Immunological factors*					
Time since HIV diagnosis (months)	168 (116–219)	205 (147–263)	–	–	0.413
CD4 count	689 (537–842)	656 (506–807)	1197 (986–1408)*	822 (711–932)	< 0.001
CD8 count	885 (748–1023)	1003 (737–1270)	485 (436–735)*	446 (364–528)*	< 0.001
Nadir CD4	188 (114–262)	219 (118–320)	–	–	0.608
HIV RNA (copies/mL)	28 (15–42)	29 (16–43)	–	–	0.845
*Medication (current)*					
HIV treatment duration (months)	130 (97–164)	125 (87–164)	–	–	0.835
Lipid lowering drug (%)	72	6	58	16	< 0.001
Insulin (%)	24	–	6	–	0.129
Metformin (%)	67	–	68	–	0.909
GLP-1 (%)	6	–	26	–	0.087
DPP-4 (%)	11	–	11	–	0.954
Sulphonylureas (%)	28	–	21	–	0.634
Special diet (%)	6	–	21	–	0.168

Differences between groups were analyzed using one-way ANOVA followed by t-test for comparison between HIV + DM2+ and the 3 control groups when ANOVA test was < 0.05. *p ≤ 0.05 compared to HIV + DM2+. For categorical data Pearson chi-square test was used. Results are given as mean (95% CI) if not otherwise stated. Participants could receive more than one type of drug for diabetes treatment and can be included in more than one treatment category except in the diet group. Participants were only registered as diet treated if this was the only treatment they received for their type 2 diabetes. Abbreviations: *HIV + DM2+* Persons living with HIV with diabetes mellitus type 2, *HIV + DM2-* Persons living with HIV without diabetes mellitus type 2, *BMI* Body mass index, *Fasting-PG* Fasting plasma glucose, *Hba1c* Hemoglobin A1c, *HDL* High density lipoprotein, *LDL* Low density lipoprotein, *HOMA-IR* Homeostatic Model Assessment of Insulin Resistance

### Fat distribution

HIV + DM2+ had a non-statistically significant tendency towards more total fat measured in kg and percentages compared to HIV + DM2-(*p* = 0.059 and *p* = 0.073, respectively) and towards more trunk fat mass (*p* = 0.057 and *p* = 0.063, respectively) (Table 2).

**Table 2:** Fat distribution

	HIV + DM2+	HIV + DM2-	Controls with DM2	Healthy controls	*P*
Total fat (kg)	25.5 (19.8–31.0)	18.8 (14.1–23.4)	28.9 (25.8–32.0)	21.6 (19.3–24.0)	0.002
Trunk fat (kg)	17.2 (13.5–20.9)	12.4 (8.9–15.9)	18.6 (16.2–21.0)	13.0 (11.5–14.6)*	0.002
Limb fat (kg)	7.5 (5.5–9.5)	5.7 (4.1–7.2)	9.5 (8.4–10.5)	7.9 (6.7–9.1)	0.004
Total fat (%)	28.8 (24.4–33.3)	23.4(19.1–27.7)	34.0 (31.5–36.6)*	28.5 (25.8–31.2)	0.001
Trunk fat (%)	34.3 (29.7–38.8)	28.1 (23.0–33.2)	39.4 (36.8–42.0)*	33.5 (30.9–36.2)	0.001
Limb fat (%)	46.6 (36.3–57.0)	36.5 (28.6–44.5)	58.0 (51.6–64.5)	47.6 (41.4–53.8)	0.002
Trunk fat mass/limb fat mass ratio	2.58 (2.18–2.97)	2.34(1.84–2.85)	2.01 (1.77–2.26)*	1.74 (1.55–1.93)*	0.001

Differences between groups were analyzed using one-way ANOVA followed by t-test for comparison between HIV + DM2+ and the 3 control groups. * *p* ≤ 0.05 compared to HIV + DM2+. Abbreviations: *HIV + DM2+* Persons living with HIV with diabetes mellitus type 2, *HIV + DM2-* Persons living with HIV without diabetes mellitus type 2

No difference in limb fat mass measured in kg and percentages was found between HIV + DM2+ and HIV + DM2- (*p* = 0.130 and *p* = 0.111). Furthermore, no difference in trunk/ limb fat ratio was found between HIV + DM2+ and HIV + DM2- (*p* = 0.444) (Table 2).

We found comparable total fat mass, trunk fat mass and limb fat mass when measured in kg in HIV + DM2+ and controls with DM2 (*p* = 0.495, *p* = 0.254, and *p* = 0.068, respectively). However, HIV + DM2+ had low percentages of total fat mass, trunk fat mass and limb fat mass than controls with DM2(*p* = 0.038, *p* = 0.042 and *p* = 0.054, respectively) without however reaching statistical significance in the latter. Also, HIV + DM2+ had a higher trunk /limb fat ratio than controls with DM2 (*p* = 0.013) (Table 2).

**Fig. 1:**
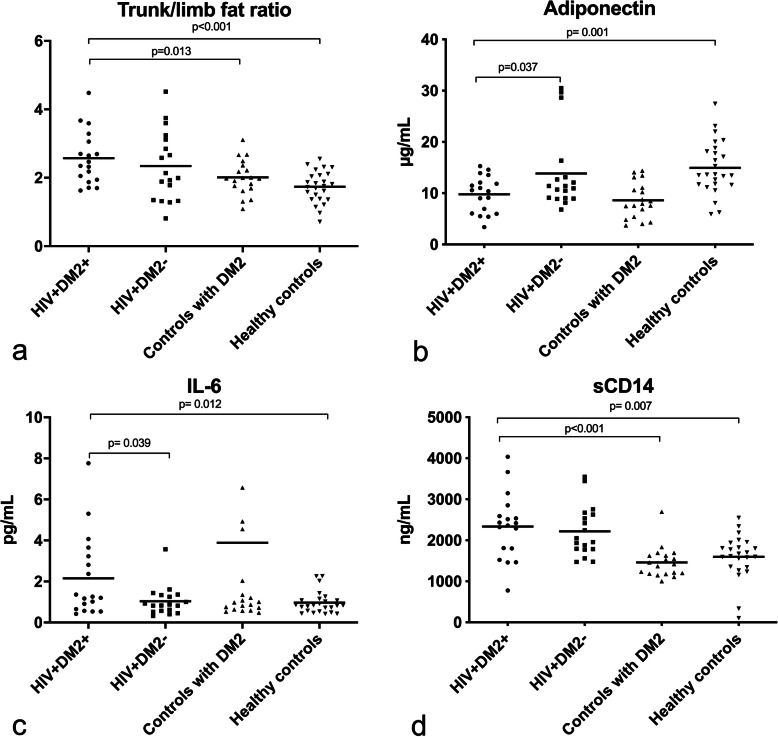
**a-d** Differences between groups were analyzed using one-way ANOVA followed by t-test. Soluble markers were log-transformed. Data shown before log-transformation. Abbreviations: HIV + DM2+; People living with HIV with diabetes mellitus type 2, HIV + DM2-; People living with HIV without diabetes mellitus type 2, IL-6; interleukin-6, sCD14; Soluble CD14

HIV + DM2+ and the healthy controls had comparable total fat mass measured in kg and percentages (*p* = 0.157 and *p* = 0.887, respectively). However, HIV + DM2+ had more trunk fat mass than the healthy controls when measured in kg but not in percentages (*p* = 0.020 and *p* = 0.746, respectively). No difference in limb fat mass was found between the two groups when measured in kg and percentages (*p* = 0.685 and *p* = 0.855, respectively). HIV + DM2+ had a higher trunk /limb fat ratio when compared to healthy controls (*p* < 0.001) (Table 2 and Fig. 1.a).

### Adiponectin

HIV + DM2+ had lower plasma adiponectin than HIV + DM2- and healthy controls (*p* = 0.037 and *p* = 0.001, respectively). In contrast, HIV + DM2+ had comparable concentration of plasma adiponectin to controls with DM2 (*p* = 0.345) (Table 3).

**Table 3:** Soluble markers

	HIV + DM2+	HIV + DM2-	Controls with DM2	Healthy controls	*P*
Adiponectin (μg/mL)	9.79 (8.02–11.56)	13.83 (10.07–17.06)*	8.63 (6.96–10.29)	14.96 (12.80–17.11)*	< 0.001
IL-6 (pg/mL)	2.16 (1.16–3.15)	1.04 (0.67–1.41)*	3.89 (0.89–8.66)	0.91 (0.72–1.11)*	0.050
TNF-alfa (pg/mL)	3.24 (2.66–3.82)	2.92 (2.01–3.84)	4.02 (1.32–6.73)	2.29 (2.07–2.51)	0.112
sCD14 (ng/mL)	2333 (1935–2732)	2218 (1912–2525)	1461 (1277–1644)*	1504 (1302–1706)*	< 0.001

Soluble markers presented as mean (95%CI) before log transformation. *P*-value represent one-way ANOVA analyses after log transformation followed by t-test for comparison between HIV + DM2+ and the 3 control groups when ANOVA test was *p < 0.05 compared to HIV+DM2+

Abbreviations: *HIV + DM2+* People living with HIV with diabetes mellitus type 2, *HIV + DM2-* People living with HIV without diabetes mellitus type 2, *IL-6* Interleukin-6, *TNF- α* Tumor necrosis factor-alfa, *sCD14* Soluble CD14, *DM2* Diabetes mellitus type 2

In the total population plasma adiponectin was negatively correlated with both trunk fat mass (kg) and trunk fat/ limb fat ratio (*r* = − 0.301, *p* = 0.007 and *r* = − 0.541, *p* < 0.001, respectively). Furthermore, plasma adiponectin was negatively correlated with HOMA-IR (*r* = − 0.464, *p* < 0.001).

### IL-6 and TNF-α

HIV + DM2+ had higher concentration of IL-6 compared to HIV + DM2- and healthy controls (*p* = 0.039 and *p* = 0.012, respectively). However, IL-6 concentration was comparable in HIV + DM2+ and controls with DM2 (*p* = 0.825). Furthermore, in the total population IL-6 was positively correlated with both trunk fat mass and HOMA-IR (*r* = 0.253, *p* = 0.024 and *r* = 0.287, *p* = 0.013, respectively).

No difference in TNF-*α* was found between the groups (Table 3).

### sCD14

HIV + DM2+ and HIV + DM2- had comparable concentration of sCD14 (*p* = 0.850). However, HIV + DM2+ had higher concentration of sCD14 compared to both controls with DM2 and healthy controls (*p* < 0.001 and *p* = 0.007, respectively) (Table 3).

### HIV status, DM2 status and association to fat distribution and soluble markers

To adjust for potential confounders and formally test for interactions between HIV infection and DM2 we performed a multiple linear regression model including HIV status, DM2 status, age and sex. Furthermore, BMI was included when one of the soluble markers were used as outcome (Tables 4 and 5).

**Table 4:** Association of HIV status and DM2 status with fat distribution

	Total fat mass			Trunk fat mass			Limb fat mass			Trunk fat mass/ limb fat mass		
	Unadjusted Β-coefficient (95% CI)	Adjusted Β-coefficient (95%CI)	Unadjusted p-interaction	Unadjusted Β-coefficient (95% CI)	Adjusted Β-coefficient (95%CI)	Unadjusted p-interaction	Unadjusted Β-coefficient (95% CI)	Adjusted Β-coefficient (95%CI)	Unadjusted p-interaction	Unadjusted Β-coefficient (95% CI)	Adjusted Β-coefficient (95%CI)	Unadjusted p-interaction
HIV status	−2.7 (−6.59;1.19)	−3.38 (−7.11;0.34)	0.882	−0.63 (−3.39;2.13)	−1.46 (−4.02;1.10)	0.758	−2.01 (− 3.39;-0.63)	−1.85 (− 3.19;-0.51)	0.825	0.60 (0.29;0.91)	0.46 (0.18;0.75)	0.905
DM2 status	6.77 (3.14;10.40)	7.17 (3.47;10.88)		5.11 (2.59;7.63)	5.65 (3.10;8.20)		1.53 (0.12;2.94)	1.39 (0.05;2.72)		0.29 (−0.04;0.63)	0.37 (0.09;0.66)	

Multivariable models have been adjusted for age and sex

Abbreviations: *DM2* Diabetes mellitus type 2

**Table 5:** Association of HIV status and DM2 status with soluble markers

	ln (Adiponectin)			ln (IL-6)			ln (TNF- *α*)			ln (sCD14)		
	Unadjusted Β-coefficient (95% CI)	Adjusted Β-coefficient (95%CI)	Unadjusted p-interaction	Unadjusted Β-coefficient (95% CI)	Adjusted Β-coefficient (95%CI)	Unadjusted p-interaction	Unadjusted Β-coefficient (95% CI)	Adjusted Β-coefficient (95%CI)	Unadjusted p-interaction	Unadjusted Β-coefficient (95% CI)	Adjusted Β-coefficient (95%CI)	Unadjusted p-interaction
HIV status	−0.03 (− 0.24;0.18)	0.04 (− 0.16;0.24)	0.180	0.08 (− 0.28;0.45)	0.06 (− 0.32;0.44)	0.886	0.13 (− 0.07;0.32)	0.19 (0.04;0.35)	0.720	0.46 (0.26;0.65)	0.44 (0.22;0.66)	0.835
DM2 status	− 0.45 (− 0.64;-0.27)	− 047 (− 0.69;-0.26)		0.51 (0.16;0.85)	0.51 (0.09;0.92)		0.21 (0.02;0.40)	0.09 (− 0.08;0.26)		0.07 (− 0.15; 0.29)	0.08 (− 0.15;0.31)	

Multivariable models have been adjusted for age, sex and BMI. Abbreviations: *IL-6* Interleukin-6, *TNF- α* Tumor necrosis factor-alfa, *sCD14* Soluble CD14, *DM2* Diabetes mellitus type 2

#### Fat distribution

In the unadjusted model HIV infection was associated with less limb fat mass (*p* = 0.005) and with higher trunk/limb fat ratio (*p* < 0.001). This was consistent after adjusting (*p* = 0.008 and *p* = 0.002, respectively).

In the unadjusted model DM2 was independently associated with more total fat mass (kg) (*p* < 0.001), trunk fat mass (kg) (*p* < 0.001) and limb fat mass (kg) (*p* = 0.037). This was consistent after adjusting (*p* < 0.001, *p* < 0.001 and *p* = 0.045, respectively) (Table 4).

#### Soluble markers

In the unadjusted model HIV infection was associated with higher concentration of sCD14 (*p* < 0.001) which was consistent after adjusting (*p* < 0.001). HIV infection was not associated with TNF-α in the unadjusted model but after adjusting HIV infection was associated with TNF-α (*p* = 0.018).

In the unadjusted model DM2 was associated with lower concentration of plasma adiponectin (*p* < 0.001) and higher concentration of IL-6 (*p* = 0.005) and TNF-α (*p* = 0.033). After adjusting DM2 was still associated with lower concentration of plasma adiponectin (*p* < 0.001) and higher concentration of IL-6 (*p* = 0.020) (Table 5).

### Interactions

No interactions were found between HIV infection and DM2 in an unadjusted model for any of the depending variables (Tables 4 and 5).

## Discussion

We found no evidence of a synergistic effect of HIV infection and DM2 on fat distribution or inflammatory markers in this study. Both PLWH with DM2 and controls with DM2 had low plasma adiponectin and high IL-6 concentrations. Furthermore, PLWH had higher sCD14 concentrations and high trunk/limb fat ratio compared to controls with DM2 and healthy controls.

In this study PLWH both with and without DM2 had higher trunk/limb fat ratio compared to controls with DM2 and healthy controls. Elevated trunk/limb fat ratio is often included in the clinical diagnosis of lipodystrophy in PLWH and is associated with increased risk of CVD [21, 22]. Altered fat distribution has been related to exposure to older generation cART [5]. Since PLWH included in the study had been living with HIV for median 14 years, the altered fat ratio could be related to exposure to older generation of cART.

Adiponectin is an adipokine with anti-inflammatory properties. Reduction in plasma adiponectin levels has been associated with increased CVD risk in both PLWH and uninfected controls. As reported in this systematic review and meta-analyses previous studies described lower concentration of plasma adiponectin in persons with DM2 [10]. Accordingly, lower concentration of plasma adiponectin was found in HIV + DM2+ than in HIV + DM2-. Furthermore, no difference in plasma adiponectin concentration between HIV + DM2+ and controls with DM2 and plasma adiponectin was negatively correlated with HOMA-IR in this cohort. Adiponectin is produced by adipocytes and is decreased in obesity due to dysfunctional hypertrophic adipocytes [23]. Especially, visceral fat accumulation has been associated with lower plasma adiponectin in both PLWH and in the general population which is consistent with the negative correlation between trunk fat mass and plasma adiponectin found in this study [9, 23]. HIV + DM2+ and controls with DM2 had comparable amount of trunk fat mass measured in kg, which may explain the similar concentrations of plasma adiponectin in the two groups.

As in adiponectin production, adipose tissue plays an important role in the production of IL-6 [23] and several studies have shown that obesity is associated with elevated IL-6 concentration in both PLWH as well as in the general population [15, 24]. We did hypothesize a synergistic effect of HIV infection and DM2 on IL-6 concentrations since HIV infection has also been shown to induce IL-6 production [25] and higher concentration of IL-6 are found in PLWH compared to non-infected controls [14, 24]. Contrary to our hypotheses IL-6 concentration was comparable in HIV + DM2+ and controls with DM2 and positively correlated with both HOMA-IR and trunk fat mass. We speculated that in the context of well-treated and virally suppressed PLWH, metabolic-driven inflammation may outweigh pro-inflammatory stimuli associated with chronic HIV infection. Also, different use of medication with anti-inflammatory properties between the groups could have affected our results. More HIV + DM2+ were treated with lipid lowering drugs including statins than the participants in the other groups. Besides the lipid-lowering effect statins are thought to have anti-inflammatory properties since statin use is found to reduce CRP levels and perhaps increase adiponectin in the general population [26, 27]. However, smaller studies investigating the impact of statin use on inflammatory markers in PLWH could not confirm this effect in PLWH [28, 29]. Furthermore, more controls with DM2 received glucagon-like-peptide-1 (GLP-1) analogues, although not significantly, which is also thought to have anti-inflammatory properties [30]. Thus, differences in statin and GLP-1 use between the groups may be a confounder in this study. However, due to the sample size we were not able to adjust for this in our analyses. Importantly, both low plasma adiponectin and elevated IL-6 are associated with increased risk of CVD in both PLWH and the general population [11, 15, 31, 32].

We did not find any difference in TNF-α concentrations between the groups. However, higher TNF-α concentration was associated with HIV infection in the adjusted analysis which is consistent with previous studies [13, 33].

sCD14 is a marker of monocyte activation [34] and elevated concentrations are reported in PLWH [35]. Furthermore, elevated concentration of sCD14 have been associated with increased mortality in PLWH [36]. Importantly, DM2 has been associated with microbial translocation and elevated concentrations of lipopolysaccharide (LPS), which is a strong inducer of sCD14 [37]. In the present study, we found elevated concentrations of sCD14 in PLWH both with and without DM2 compared to both controls with DM2 and healthy controls. A larger study found decreased concentration of sCD14 in HIV/obese persons compared to HIV/non-obese persons [38]. In contrast, an increase in sCD14 concentration in overweight and obese PLWH gaining weight after initiation of cART has been reported [39]. To our knowledge data on sCD14 and DM2 is still very limited both in PLWH and uninfected persons. Our data suggest that elevated concentration of sCD14 is related to HIV status and not DM2 or fat distribution.

This study is limited by its cross-sectional design and relatively small number of participants. Furthermore, it is a limitation that HIV testing was not performed consistently in all participants resulting in a risk of undiagnosed HIV in the control groups. However, the prevalence of HIV in Denmark is low (0.1%), and it seems unlikely that undiagnosed HIV is a major confounder. Also, more detailed information concerning complications to DM2 would have added valuable information to the clinical characteristics of the study population. However, the study included three control groups; PLWH without DM2, persons with DM2 and healthy controls matched on age allowing us to determine association of both HIV infection and DM2 and is to the best of our knowledge the first study to investigate the combined effect of HIV infection and DM2 on fat distribution and inflammation in this setting.

## Conclusion

In conclusion, both well-treated PLWH in the contemporary cART era and persons with DM2 had altered fat distribution and chronic inflammation. However, no evidence of a synergistic effect of HIV infection and DM2 on fat distribution, plasma adiponectin or inflammatory markers was found. However, our data suggest that HIV is associated with higher trunk/limb fat ratio, higher concentration of sCD14 and possible higher concentration of TNF-α. Furthermore, DM2 was associated with increased trunk fat mass, low concentration of plasma adiponectin, and high concentration of IL-6, probably driven in part by increased trunk fat mass. Thus, persons living with both diseases may have a higher inflammatory state driven by both fat tissue and HIV induced immune activation. This was an exploratory study with a limited power, but it points to markers that should be further investigated to gain knowledge about the mechanisms and risk factors related to the increased risk of CVD in PLWH with DM2.

## Data Availability

The dataset analysed during the current study is available from the corresponding author on reasonable request.

## Abbreviations

CVD: Cardiovascular disease
cART: Combination antiretroviral treatment
DM2: Diabetes mellitus type 2
DXA–scan: Dual-energy X-ray absorptiometry scan
HOMA-IR: Homeostatic Model Assessment of Insulin Resistance (HOMA-IR)
IL-6: Interleukin-6
PLWH: Persons living with HIV
HIV + DM2+: Persons living with HIV with DM2
HIV + DM2-: Persons living with HIV without DM2
sCD14: Soluble CD14
TNF- *α*: Tumor necrosis factor-alfa
